# Polar Metallocenes

**DOI:** 10.3390/molecules24030486

**Published:** 2019-01-29

**Authors:** Haiwu Zhang, B.Yu. Yavorsky, R.E. Cohen

**Affiliations:** 1 Department of Earth and Environmental Sciences, Ludwig-Maximilians Universität München, Theresienstr, 41 80333 Munich, Germany; zhw3789@sina.com (H.Z.); bogdan.yavorskyy@o2online.de (B.Y.Y.); 2 Extreme Materials Initiative, Geophysical Laboratory, Carnegie Institution for Science, Washington, DC 20015, USA

**Keywords:** metallocenes, DFT, vdW interactions, polarization, materials design

## Abstract

Crystalline polar metallocenes are potentially useful active materials as piezoelectrics, ferroelectrics, and multiferroics. Within density functional theory (DFT), we computed structural properties, energy differences for various phases, molecular configurations, and magnetic states, computed polarizations for different polar crystal structures, and computed dipole moments for the constituent molecules with a Wannier function analysis. Of the systems studied, Mn_2_(C_9_H_9_N)_2_ is the most promising as a multiferroic material, since the ground state is both polar and ferromagnetic. We found that the predicted crystalline polarizations are 30–40% higher than the values that would be obtained from the dipole moments of the isolated constituent molecules, due to the local effects of the self-consistent internal electric field, indicating high polarizabilities.

## 1. Introduction

Metallocenes have manifold uses ranging from fire retardants, catalysts, and cancer pharmaceuticals [1,2,3]. Almost all applications of metallocenes are for their molecular properties; however, here we consider crystalline metallocenes and their potential as active, multiferroic, materials.

The discovery of ferrocene, which has two cyclopentadienyl (Cp, or C_5_H_5_^−^) rings with a transition metal sandwiched between, was the beginning of the field of organometallic chemistry, with the 1973 Nobel prize awarded to Geoffrey Wilkinson and Ernst Otto Fisher [4,5]. There are many possible metallocenes, since the charge of the cyclic conjugated ligands -C_n_H_n_^q^ (*n* = 3–8) can be positive, negative, or neutral [6]; the hydrogen atom(s) can be replaced by isovalent functional groups (-X, X = F, Cl, Br, I, OH, CH_3_, etc.) [7,8,9,10]; the -C_5_H_5_^−^ ring can be replaced by other heterocycles; the Fe cation can be replaced by other transition metals with different spin states [11,12,13], and metallocene molecules can be doubled [14] or coordinated by other molecules or ligands. Here, we explored building polar crystals through the crystallization of polar molecules. Ferrocene itself has no (when the rings thermally rotate) or only a small possible dipole moment (as a static molecule) since it is a symmetric molecule. It crystallizes in a monoclinic structure (P2_1_/a) at room temperature [15], which transforms metastably to triclinic at 164 K [16], or stably to orthorhombic [17]. The centrosymmetric staggered molecular structure within the P2_1_/a phase transfers into two slightly distinct types of non-centrosymmetric, almost eclipsed, ferrocene molecules within the triclinic phase [16,18,19]. Density functional theory (DFT) calculations show that the ferrocene molecule with a staggered configuration has a very weak dipole moment (0.006 D), and the two types of molecules within the triclinic crystalline ferrocene increase to 0.08 D and 0.04 D, respectively [20]. Recently, using first-principle calculations, Wu et al. [21] predicted that crystallized M(C_6_H_6_) and M(C_5_H_5_) nanowires become ferroelectric or even multiferroic, depending on the decorating functional groups. Nanowires of M(Cp)∞ (M = Ti, Cr, Fe) are predicted to be ferromagnetic half-metals, and [M(FeCp_2_)]∞ (M = Sc, Ti, V, Mn) are ferromagnetic semiconductors [22,23].

## 2. Assessing van der Waals (vdW) Functionals

Density functional theory gives reasonable geometries for metallocene molecules [24,25,26]. Xu et al. [27] compared several gradient-corrected functionals, as well as the local density approximation (LDA), and found that the B3LYP functional gives the best agreement between the experimental and theoretical molecular geometry. However, for molecular crystals, accurate van der Waals forces must be included. We computed the equilibrium crystalline geometry starting from the experimental room temperature structure, the magnetic ground states, and magnetic moments of ferrocene, nickelocene, cobaltocene, and vanadocene, which are experimentally available, to assess the performance of the vdW functionals DFT-D, rVV10, and vdW-DF2 [28,29,30].

We found that the vdW-DF2 functional is most accurate of the three (Table 1), and provides quite good agreement with experiments, within ~3% of the observed cell volumes. In contrast, DFT-D and rVV10 methods significantly underestimate cell volumes. Therefore, we employed the vdW-DF2 functional for the subsequent calculations.

## 3. Dipole Moments for Isolated Molecules

**Figure 1:**
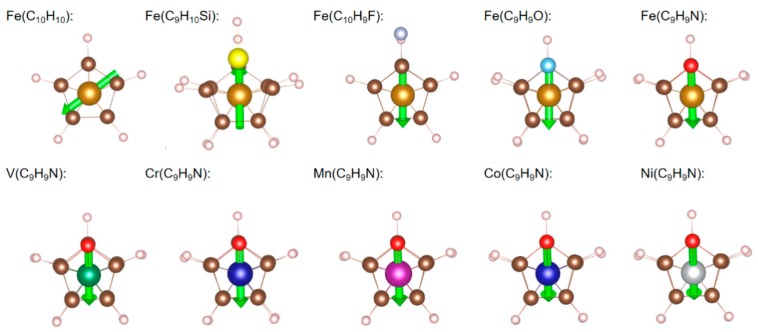
The structure and dipole moments of isolated Fe(C_10_H_10_), Fe(C_9_H_10_Si), Fe(C_10_H_9_F), Fe(C_9_H_9_O), Fe(C_9_H_9_N), V(C_9_H_9_N), Cr(C_9_H_9_N), Mn(C_9_H_9_N), Co(C_9_H_9_N), and Ni(C_9_H_9_N) molecules with an eclipsed starting configuration. The green arrows denote the direction of the dipole moment for each molecule. Key: C: brown; Fe: shallow brown; H: pink; O: light blue; N: red; V: cyan; Cr: dark blue; Mn: purple; Co: blue; Ni: silver.

Next, we considere maximizing the dipole moments for isolated molecules. We relaxed the isolated molecules in periodic cells of various sizes, to compare directly with our plane wave pseudopotential computations using the same basis set for crystals [32]. We computed the dipole moments using maximally localized Wannier functions [33]. We found that the dipole moment of an isolated ferrocene molecule with an eclipsed configuration is 0.08 D, with the direction being parallel to the two Cp rings (Figure 1, Table 2), consistent with simulation results of J. M. B.-García, et al. [20]. The molecular dipole moments can be increased by ligand decorations or by replacing the Cp with heterocyclic rings. The replacing of one C atom by Si increases the dipole moment substantially to 0.25 D for Fe(C_9_H_10_Si), with the positive polarization towards Si and the unsubstituted Cp ring. This is consistent with the weaker electronegativity of Si relative to C. When we replaced one H atom by F, we found more electrons on the -C_5_H_4_F^−^ ring than on the Cp side, consistent with the stronger electronegativity of F with respect to H. Therefore, Fe(C_10_H_9_F) has a large dipole moment (1.70 D), pointing away from the unsubstituted Cp ring. Experimentally, metallocenes with 1–5 halogens per ring have been synthesized [10,34,35,36,37,38,39]. When we replaced F with heavier halogens, we found that the dipole moment increased to 1.77, 1.82, and 1.81 D in Fe(C_10_H_9_Cl), Fe(C_10_H_9_Br), and Fe(C_10_H_9_I), respectively (Table 3). We propose that the increase in dipole moment was simply due to the larger bond lengths of the positive and negative charges, opposite to what one would expect from electronegativity, which decreases with increasing atomic number.

Next, we considered replacing a -C_5_H_5_^−^ ring by a charge neutral furan (-C_4_H_4_O). This sandwich structure was synthesized experimentally for the Ru-metallocene [40]. The iron atom donated one less electron to the neutral -C_4_H_4_O ring, giving rise to Fe^+^ (d^7^), and 19 electrons for the molecule. We obtained, for furanferrocene Fe(C_9_H_9_O), a magnetic moment of 1 μB (indicating a low-spin iron), and a large dipole moment (1.32 D). When we replaced a Cp ring with pyrrole (-C_4_H_4_N^−^), we found a larger dipole moment (2.08 D). The dipole moment of Fe(C_9_H_9_N) points from the N atom to the other side of the heterocycle and to the unsubstituted Cp ring. The electron-donating capacity of B, P, As, Sb, and Bi are weaker than that of N; however, the dipole moment decreases to 0.87, 1.71, 1.42, 0.70 and 0.52 D in Fe(C_9_H_9_B), Fe(C_9_H_9_P), Fe(C_9_H_9_As), Fe(C_9_H_9_Sb), and Fe(C_9_H_9_Bi), respectively (Table 4).

The magnitude of the dipole moment of azametallocene molecules shows a strong dependence on the atomic number of the cations (Table 2). Similar to M_2_(C_10_H_10_)_2_, the cations in azametallocene molecules donate two electrons (one to -C_5_H_5_^−^ and the other to -C_4_H_4_N^−^) to form a stable aromatic configuration having 4k + 2 (k is an integer) electrons [41]. We found for vanadium in V(C_9_H_9_N) that the most stable 3d electronic configuration is the one with three parallel spins. Iron in Fe(C9H9N) is in a low spin state with zero magnetic moment. These results agree well with TM_2_(C_10_H_10_)_2_ (Table 1), as the substitution of -C_5_H_5_^−^ by -C_4_H_4_N^−^ does not change the number of electrons on the cations.

The relative stabilities and dipole moments of isolated metallocene molecules with two ring rotations show low rotation barriers (*E*_rotation_ = *E*_staggered_ − *E*_eclipsed_), which are lower than the rotation barrier within the crystalline ferrocene (4.4 ± 0.5 kJ/mol), [42] and comparable to ~*k*_B_T at room temperature (~2.6 kJ/mol, Table 2, Table 3 and Table 4). Therefore, the rotation of the Cp rings is unhindered at room temperature. The negative values of *E*_rotation_ for Fe(C_9_H_9_O) and Ni(C_9_H_9_N) suggest that the staggered configuration, rather than the eclipsed configuration, is the ground state. Furthermore, we found that the dipole moments are dependent on the relative rotation of the rings. Upon rotation from the ground state to the transition state, the dipole moments vary by 10%, suggesting that ring rotation can be activated by applying time varying electric fields.

## 4. Crystalline Polar Metallocenes

**Figure 2:**
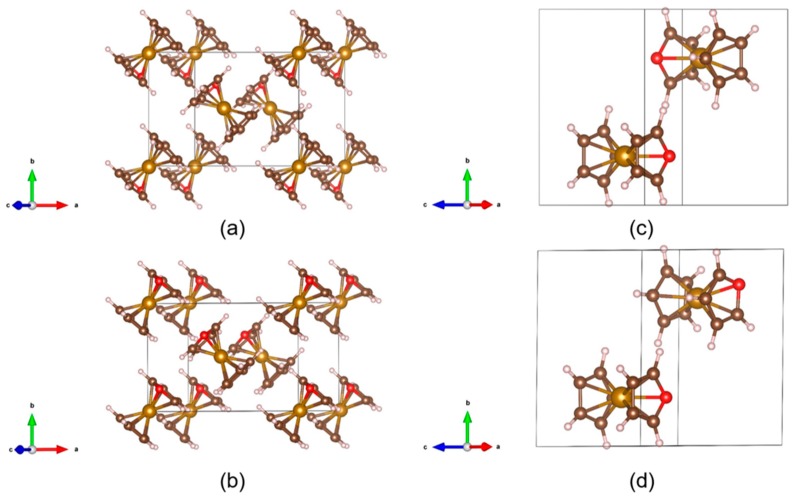
Optimized crystal structure of azaferrocene constructed using the (**a**) room temperature and (**b**) low temperature monoclinic ferrocenes as prototype structures; triclinic ferrocene with the two constituent azaferrocene molecules (**c**) antiparallel (AFE) and (**d**) almost parallel (FE) to each other. Azaferrocene constructed using the two monoclinic ferrocene structures has a polar ground state. The two structures are denoted as M1 and M2, respectively. Key: C: dark brown; Fe: brown; H: pink; N: red.

Returning to crystalline properties, we considere polar metallocenes composed of molecules that display promising dipole moments. Azaferrocene Fe_2_(C_9_H_9_N)_2_ is isomorphous with ferrocene and crystallizes at room temperature in the monoclinic structure [43,44]. Thus, we first constructed the initial monoclinic azaferrocene using the room temperature [15] and low temperature [45] monoclinic ferrocene as prototype structures. They were found to be polar at their ground state, as shown in Figure 2a (M1) and b (M2), respectively. The net polarization depends on the magnitude as well as the arrangement of the dipole moments of the two constituent molecules in the unit cell. Therefore, we further consider two extreme cases, namely, the two constituent molecules antiparallel (AFE) and almost parallel (FE) to each other, relaxed from the triclinic ferrocene structure [10]. Within the AFE configuration, the dipole moments of the two molecules cancel each other, giving rise to a zero polarization (Figure 2c). The almost parallel arrangement of the dipole moments within the FE configuration yields the largest polarization among various structures investigated (Figure 2d).

The energy differences of the AFE configuration with respect to the FE configuration (Δ*E*_polar_ = *E*_FE_ − *E*_AFE_, meV/per cell) are also shown in Table 5 to evaluate their relative stabilities. Mn_2_(C_9_H_9_N)_2_ has a negative Δ*E*_polar_, which is highly desirable as the FE configuration is the ground state. All the other systems investigated have an AFE configuration. To investigate the stability of the crystals, we calculated the binding energy by *E*_bind_ = *E*_molecule_ − *E*_ground_/2, where *E*_molecule_ is the energy of the isolated molecules at their ground state, and *E*_ground_ is the total energy per unit cell of triclinic polar metallocenes with the FE configuration at their magnetic ground state. The binding energies are large, ranging from 0.57 to 0.91 eV, and the relaxed cell volumes of azametallocenes decrease as the computed binding energies increase. The low energy differences between the AFE and FE configuration together with the large binding energies, suggest that such systems with ferroelectric ordering can be fabricated by applying an external bias field during crystallization and could be stable over a wide temperature range.

We further investigated the magnetic and electronic properties of polar metallocenes with the FE configuration. We computed the energy differences between the antiferromagnetic and ferromagnetic states (Δ*E*_MS_ = *E*_AFM_ − *E*_FM_) to estimate the exchange splitting, as shown in Table 6. Like ferrocene, Fe_2_(C_10_H_9_F)_2_ and Fe_2_(C_9_H_9_N)_2_ are non-magnetic. Fe_2_(C_9_H_9_O)_2_ has an AFM ground state. Similar to V_2_(C_10_H_10_)_2_ and Ni_2_(C_10_H_10_)_2_ (Table 1), we found that V_2_(C_9_H_9_N)_2_, Cr_2_(C_9_H_9_N)_2_, and Ni_2_(C_9_H_9_N)_2_ have AFM ground states, which are more stable than the FM states by 6.19, 1.60, and 4.93 meV per unit cell, respectively. Unlike Co_2_(C_10_H_10_)_2_, Co_2_(C_9_H_9_N)_2_ has a FM ground state, and has the largest Δ*E*_MS_ (47.57 meV) among different azametallocenes investigated. Similarly, Mn_2_(C_9_H_9_N)_2_ has a FM ground state with a quite small energy difference (0.47 meV). Therefore, Mn_2_(C_9_H_9_N)_2_ is the most promising as ferroic material, since FM and FE ordering can be realized simultaneously.

With an FE configuration, the metallocene crystals formed from molecules with large dipole moments show large polarizations. The large polarizations observed for azametallocenes (e.g., >5 μC/cm^2^, Table 6) with respect to Fe_2_(C_10_H_9_F)_2_ and Fe_2_(C_9_H_9_O)_2_, are due to the large dipole moments of the component molecules. Similar to the phenomena observed for water [46,47], the polarization for the crystalline phase is about 30–40% higher than the values obtained based on the dipole moments for isolated molecules. This is due to the polarization of the molecules by the local self-consistent electrical field, and indicates high polarizabilities. Although the polarization of azametallocenes is smaller than inorganic perovskites (e.g., ~30 μC/cm^2^ for BaTiO_3_ [48]), a large piezoelectric response is possible, since metallocenes rae mechanically softer.

## 5. Materials and Methods

All the calculations were performed using the pseudopotential plane-wave method implemented in the Quantum Espresso code [32]. Three different van der Waals functionals, namely, DFT-D, rVV10, and vdW-DF2, were incorporated to describe the van der Waals interactions [49,50,51,52,53,54]. The van der Waals interaction is part of the correlation energy, and can be applied to the different exchange functionals. In particular, in Quantum Espresso, the non-local functionals vdW-DF2 and rVV10 are used together with the revised Perdew-Wang functional (rPW86) [55,56], and the DFT-D correction was applied to the generalized gradient approximation of Perdew, Burke, and Ernzerhof (PBE-GGA) [57].

The lattice constants as well as atomic positions are optimized until the forces were smaller than 0.01 eV/Å. The kinetic energy cutoffs for wavefunctions and charge density were set to 544.22 (40) and 2721.13 (200) eV(Ry), respectively. For crystalline metallocenes, the Brillouin zone was sampled by the Monkhorst-Pack scheme (4 × 4 × 4 k points for structure optimization, 6 × 6 × 6 k points for the subsequent self-consistent and property calculations). The ‘isolated molecules’ were simulated by putting the molecules into fixed unit cells with different sizes. We found unit cells with a size of 24 × 24 × 24 Å^3^ were large enough to well describe the internal stress and dipole moment of the isolated molecules. Maximally localized Wannier functions were generated by the Wannier90 code [33] to determine the negative charge center, based on which the dipole moments of the isolated molecules were calculated. GBRV pseudopotentials were employed for all the calculations [58].

## 6. Conclusions

We found polar metallocenes to be a worthwhile group to study as new active materials. Only recently has first-principles study of these systems become possible with new local van der Waals functionals, which we found to be indispensable. The vdW-DF2 functional gives accurate predictions for the structures and magnetic ground states of metallocene molecules and crystals. The dipole moment of isolated ferrocene molecules could be effectively tuned by ligand decorations or by replacing the Cp with heterocyclic rings. The magnetic moment of the ferrocene molecules could also be tuned by changing the charge of the cyclic conjugated ligands and/or by substitution of iron with other cations that had a different spin state. Our results suggest that crystalline polar metallocenes are worthy of study as potential ferroelectrics and multiferroics.

## Figures and Tables

**Table 1:** Static properties for ordered crystalline metallocenes. Results for magnetic order (FM, AFM and NM denote ferromagnetic, antiferromagnetic and non-spin-polarized, respectively), energy difference (Δ*E*_MS_, eV/molecule) relative to magnetic ground state, magnetic moment (*M*, μB/molecule), and cell volume (*V*_cell_, Å^3^), of ferrocene, nickelocene, cobaltocene, and vanadocene, calculated using DFT-D, rVV10, and vdW-DF2 functionals. For ferrocene, a starting configuration with AFM or FM converges to the non-magnetic state for all three functionals. Therefore, the magnetic moment for the FM state is fixed to 8.0 μB per unit cell for comparison. Note that Ni_2_C_20_H_20_ with an AFM starting configuration also converges to non-magnetic with the rVV10 functional.

		DFT-D			rVV10			vdW-DF2			Exp
		FM	AFM	NM	FM	AFM	NM	FM	AFM	NM	
Fe_2_(C_10_H_10_)_2_	Δ*E*_MS_	2.222	-	0.00	1.871	-	0.00	1.55	-	0.00	-
	*M*	4.05	-	0.00	4.05	-	0.00	4.05	-	0.0	0.00 ^a^
	*V*_cell_	366.43	-	344.37	387.12	-	363.45	416.37	-	391.85	395.00 ^b^
Ni_2_(C_10_H_10_)_2_	Δ*E*_MS_	0.0	0.001	0.310	0.00	-	0.365	0.00	0.037	0.166	-
	*M*	2.18	2.15	0.00	2.18	-	0.00	2.17	2.15	0.00	2.89 ^a^
	*V*_cell_	358.10	358.23	353.87	373.25	-	370.79	398.08	384.73	412.09	403.67 ^b^
Co_2_(C_10_H_10_)_2_	Δ*E*_MS_	1.181	0.00	0.225	0.948	0.00	1.432	0.755	0.00	0.468	-
	*M*	3.11	1.24	0.00	3.11	1.25	0.00	3.10	1.25	0.00	1.73 ^a^
	*V*_cell_	366.09	351.22	350.59	386.45	371.93	450.32	421.09	405.40	462.36	418.57 ^b^
V_2_(C_10_H_10_)_2_	Δ*E*_MS_	0.003	0.00	1.200	0.003	0.00	1.252	0.001	0.000	1.104	-
	*M*	3.11	3.11	0.00	3.09	3.18	0.00	3.16	3.08	0.00	3.78 ^a^
	*V*_cell_	370.57	369.95	356.99	387.93	387.48	376.42	418.79	418.54	418.97	414.77 ^b^

^a^ Reference [31]; ^b^ References [15,28,29,30], for ferrocene, nickelocene, cobaltocene, and vanadocene, respectively.

**Table 2:** Number of 3*d* electrons on the transition metal (TM) ions (N); magnetic moment (M, μB), dipole moment (μ, D) of isolated metallocene molecules; rotation barriers (*E_rotation_* = *E_staggered_* − *E_eclipsed_*, kJ/mol) and dipole moment differences (Δ*μ* = *μ_staggered_* − *μ_eclipsed_*, D) between the two different configurations.

	Fe(C_10_H_10_)	Fe(C_9_SiH_10_)	Fe(C_10_H_9_F)	Fe(C_9_H_9_O)	Fe(C_9_H_9_N)	V(C_9_H_9_N)	Cr(C_9_H_9_N)	Mn(C_9_H_9_N)	Co(C_9_H_9_N)	Ni(C_9_H_9_N)
*N*	6	6	6	7	6	3	4	5	7	8
*M*	0	0	0	1	0	3	2	1	1	2
*μ*	0.08	0.25	1.70	1.32	2.08	1.75	2.37	1.84	2.20	2.80
*E_rotation_*	2.61	3.38	2.41	−3.76	2.22	0.29	0.97	2.12	1.06	−1.74
Δ*μ*	−0.01	0.04	0.04	0.07	−0.25	0.31	−0.40	0.26	0.02	−0.03

**Table 3:** Dipole moment (*μ*, D) of isolated metallocene molecules with an eclipsed configuration; activation energy (*E*_act_ = *E*_transition state_ − *E*_ground state_, kJ/mol) and dipole moment difference (Δ*μ* = *μ*_transition state_ − *μ*_ground state_, D) between the transition state and the ground state. The number of 3*d* electrons on Fe is 6, and the magnetic moment of each isolated molecule is 0.

	Fe(C_10_H_9_Cl)	Fe(C_10_H_9_Br)	Fe(C_10_H_9_I)
*μ*	1.77	1.82	1.81
*E*_act_	2.19	2.16	2.01
Δ*μ*	0.01	−0.21	−0.02

**Table 4:** Dipole moment (*μ*, D) of isolated metallocene molecules with an eclipsed configuration; activation energy (*E*_act_ = *E*_transition state_ − *E*_ground state_, kJ/mol) and dipole moment difference (Δ*μ* = *μ*_transition state_ − *μ*_ground state_, D) between the transition state and the ground state. The number of the 3*d* electrons rest on Fe is 6, and the magnetic moment of the isolated molecules is 0.

	Fe(C_9_H_9_B)	Fe(C_9_H_9_P)	Fe(C_9_H_9_As)	Fe(C_9_H_9_Sb)	Fe(C_9_H_9_Bi)
*μ*	0.87	1.71	1.42	0.70	0.52
*E*_act_	3.41	2.60	3.60	3.08	3.19
Δ*μ*	0.10	0.01	−0.11	0.16	0.30

**Table 5:** Cell volume (*V*_cell_, Å^3^) at magnetic ground state, energy difference of the triclinic polar metallocenes with the FE configuration with respect to the AFE configuration (Δ*E*_polar_ = *E*_FE_ − *E*_AFE_, meV per cell), binding energy (*E*_bind_ = *E*_molecule_ − *E*_ground_/2, eV per molecule).

	Fe_2_(C_10_H_9_F)_2_	Fe_2_(C_9_H_9_O)_2_	V_2_(C_9_H_9_N)_2_	Cr_2_(C_9_H_9_N)_2_	Mn_2_(C_9_H_9_N)_2_	Fe_2_(C_9_H_9_N)_2_	Co_2_(C_9_H_9_N)_2_	Ni_2_(C_9_H_9_N)_2_
*V*_cell_	404.58	382.58	404.87	392.73	386.79	378.17	385.17	382.98
Δ*E*_polar_	80.96	74.96	12.22	5.99	−0.87	17.67	44.78	6.53
*E*_bind_	0.89	0.76	0.90	0.85	0.76	0.91	0.87	0.57

**Table 6:** Magnetic ground state (MGS), energy difference between AFM and FM states (Δ*E*_MS_ = *E*_AFM_ − *E*_FM_, meV/per cell), magnetic moment (*M*_cell_, μB), polarization (*P*, μC/cm^2^) by Berry’s phase calculations, and polarization (P*, μC/cm^2^) calculated by summing the dipole moment of isolated molecules for triclinic polar metallocenes with the FE configuration.

	Fe_2_(C_10_H_9_F)_2_	Fe_2_(C_9_H_9_O)_2_	V_2_(C_9_H_9_N)_2_	Cr_2_(C_9_H_9_N)_2_	Mn_2_(C_9_H_9_N)_2_	Fe_2_(C_9_H_9_N)_2_	Co_2_(C_9_H_9_N)_2_	Ni_2_(C_9_H_9_N)_2_
MGS	/	AFM	AFM	AFM	FM	/	FM	AFM
Δ*E*_MS_	/	−7.92	−6.20	−1.60	0.47	/	47.57	−4.93
*M*_cell_	0	0	0	0	2.0	0	2.0	0
*P*	4.40	3.16	5.03	6.06	5.43	5.56	5.79	6.56
P*	2.72	1.91	2.77	3.92	3.07	3.55	3.69	4.80

## References

[1] Nicolaev G.A., Nicolaev A. (2014). Transport in ferrocene single molecules for terahertz applications. Phys. Chem. Chem. Phys..

[2] Cunha T.F.D., Calderini D., Skouteris D. (2016). Analysis of Partition Functions for Metallocenes: Ferrocene, Ruthenocene, and Osmocene. J. Phys. Chem. A..

[3] Wang P., Jiang X., Hu J., Huang X., Zhao J., Ahuja R. (2017). Prediction of huge magnetic anisotropies in 5d transition metallocenes. J. Phys. Condens. Matter..

[4] Fischer O., Pfab W. (1952). Cyclopentadien-Metallkomplexe, ein neuer Typ metallorganischer Verbindungen. Z. Nat. B.

[5] Wilkinson G.R.M., Whiting M.C. (1952). The Structure of Iron Bis-Cyclopentadienyl. J. Am. Chem. Soc..

[6] Frunzke J., Lein M., Frenking G. (2002). Structures, Metal-Ligand Bond Strength, and Bonding Analysis of Ferrocene Derivatives with Group-15 Heteroligands Fe(η^5^-E_5_)_2_ and FeCp(η^5^-E_5_) (E=N, P, As, Sb). A Theoretical Study. Organometallics.

[7] Dombrowski K.E., Baldwin W., Sheats J.E. (1986). Metallocenes in biochemistry, microbiology and medicine. J. Organomet. Chem..

[8] Dunitz J.D., Orgel L.E. (1955). Electronic Structure of Metal biscyclopentadienyls. J. Chem. Phys..

[9] Vargas-Caamal A., Pan S., Ortiz-Chi F., Cabellos J.L., Boto R.A., Contreras-Garcia J., Restrepo A., Chattaraj P.K., Merino G. (2016). How strong are the metallocene–metallocene interactions? Cases of ferrocene, ruthenocene, and osmocene. Phys. Chem. Chem. Phys..

[10] Sünkel K., Weigand S., Hoffmann A., Blomeyer S., Reuter C.G., Vishnevskiy Y.V., Mitzel N.W. (2015). Synthesis and Characterization of 1,2,3,4,5-Pentafluoroferrocene. J. Am. Chem. Soc..

[11] Sohn Y.S., Hendrickson D.N., Gray H.B. (1971). Electronic Structure of Metallocenes. J. Am. Chem. Soc..

[12] Haaland A. (1979). Molecular Structure and Bonding in the 3d Metallocenes. Acc. Chem. Res..

[13] Little W.F. (1963). Metallocenes. Surv. Prog. Chem..

[14] Wu X., Zeng X. (2009). Double Metallocene Nanowires. J. Am. Chem. Soc..

[15] Seiler P., Dunitz J.D. (1979). A New Interpretation of the Disordered Crystal Structure of Ferrocene. Acta Cryst..

[16] Dunitz J.D. (1995). Phase Changes and Chemical Reactions in Molecular Crystals. Acta Cryst. B.

[17] Seiler P., Dunitz J.D. (1982). Low-temperature Crystallization of Orthorhombic Ferrocene: Structure Analysis at 98 K. Acta Cryst. Sect. B-Struct. Sci..

[18] Seiler P., Dunitz J.D. (1979). The Structure of Triclinic Ferrocene at 101, 123 and 148 K. Acta Cryst..

[19] Edwards J.W., Kington G.L., Mason R. (1960). The thermodynamic properties of ferrocene. Trans. Faraday Soc..

[20] Bermúdez-García J.M., Yáñez-Vilar S., Castro-García S., Señarís-Rodríguez M.A., Sánchez-Andújar M. (2015). New properties in old systems: Cooperative electric order in ferrocene and ammonia-borane. RSC Adv..

[21] Wu M., Burton J.D., Tsymbal E.Y., Zeng X.C., Jena P. (2012). Multiferroic Materials Based on Organic Transition-Metal Molecular Nanowires. J. Am. Chem. Soc..

[22] Shen L., Yang S.-W., Ng M.-F., Ligatchev V., Zhou L., Feng Y. (2008). Charge-Transfer-Based Mechanism for Half-Metallicity and Ferromagnetism in One-Dimensional Organometallic Sandwich Molecular Wires. J. Am. Chem. Soc..

[23] Zhang X., Wang J., Gao Y., Zeng X. (2009). Ab Initio Study of Structural and Magnetic Properties of TM*_n_*(ferrocene)*_n_*_+1_ (TM = Sc, Ti, V, Mn) Sandwich Clusters and Nanowires (*n* = ∞). ACS Nano.

[24] Fey N. (1999). Organometallic molecular modelling-the computational chemistry of metallocenes: A review. J. Chem. Technol. Biotechnol..

[25] Coriani S., Haaland A., Helgaker T., Jørgensen P. (2006). The Equilibrium Structure of Ferrocene. ChemPhysChem.

[26] Aliabad H.A.R., Chahkandi M. (2017). Comprehensive SPHYB and B3LYP-DFT Studies of Two Types of Ferrocene. Z. Anorg. Allg. Chem.

[27] Xu Z.-F., Xie Y., Feng W.-L., Schaefer H.F. (2003). Systematic Investigation of Electronic and Molecular Structures for the First Transition Metal Series Metallocenes M(C_5_H_5_)_2_ (M=V, Cr, Mn, Fe, Co, and Ni). J. Phys. Chem. A.

[28] Seller P., Duntitz J.D. (1980). The Structure of Nickelocene at Room Temperature and at 101 K. Acta Cryst..

[29] Yu M., Antipin R.B. (1993). Redetermination of the Cobaltocene Crystal Structure at 100 K and 297 K: Comparison with Ferrocene and Nickelocene. Struct. Chem..

[30] Yu M., Antipin R.B. (1996). Structure of Vanadocene in the Temperature Interval 108–357 K and the Nature of the Ring Disorder. Acta Cryst..

[31] Robertson R.E., Mcconnell H.M. (1960). The magnetic resonance properties of some sandwich compounds. J. Phys. Chem..

[32] Giannozzi P., Baroni S., Bonini N., Calandra M., Car R., Cavazzoni C., Ceresoli D., Chiarotti G.L., Cococcioni M., Dabo I. (2009). QUANTUM ESPRESSO: A modular and open-source software project for quantum simulations of materials. J. Phys. Condens. Matter.

[33] Marzari N., Mostofi A.A., Yates J.R., Souza I., Vanderbilt D. (2012). Maximally localized Wannier functions: Theory and applications. Rev. Mod. Phys..

[34] Rosenblum M., Fish R.W. (1965). A convenient synthesis of some haloferrocenes. J. Org. Chem..

[35] Bernhartzeder S., Sunkel K. (2012). Coordination chemistry of perhalogenated cyclopentadienes and alkynes. Part 30. New high-yield syntheses of monochloroferrocene and 1,2,3,4,5-pentachloroferrocene. Molecular structures of 1,2-dichloroferrocene and 1,2,3-trichloroferrocene. J. Organomet. Chem..

[36] Sunkel K., Bernhartzeder S. (2011). Coordination chemistry of perhalogenated cyclopentadienes and alkynes. XXVIII [1] new high-yield synthesis of monobromoferrocene and simplified procedure for the synthesis of pentabromoferrocene. Molecular structures of 1,2,3-tribromoferrocene and 1,2,3,4,5-pentabromoferrocene. J. Organomet. Chem..

[37] Sünkel K. (1997). Coordination chemistry of pentahalocyclopentadienyls. Chem. Ber. -Recl..

[38] Hnetinka C.A., Hunter A.D., Zeller M., Lesley M.J.G. (2004). 1,1′-Dibromoferrocene. Acta Cryst. E.

[39] Romanov A.S., Mulroy J.M., Khrustalev V.N., Antipin M.Y., Timofeeva T.V. (2009). Monohalogenated ferrocenes C_5_H_5_FeC_5_H_4_*X* (X = Cl, Br and I) and a second polymorph of C_5_H_5_FeC_5_H_4_I. Acta Cryst. C.

[40] Chaudret B., Jalon F.A. (1988). Facile preparation of π-arene complexes of ruthenium [(η^5^-C_5_Me_5_)Ru(Arene)]X including a π-pyridine and the first π-furan derivatives. J. Chem. Soc. Chem. Commun..

[41] Da H., Jin H.M., Lim K.H., Ligatchev V., Ng M.-F., Khoo K.H., Yang S.-W. (2012). Half metal semiconductor reversible switch in bimetallic sandwich molecular wire via redox reactions. Nano Energy.

[42] Gardner A.B., Howard J., Waddington T.C. (1981). The dynamics of ring rotation in ferrocene, nickelocene and ruthenocene by incoherent quasi-elastic neutron scattering. Chem. Phys..

[43] Brock C.P., Fu Y. (1997). Rigid-Body Disorder Models for the High-Temperature Phase of Ferrocene. Acta Cryst..

[44] Clec’h G., Calvarin G. (2011). Etude Structurale Des Phases Desordonnees De Co(C_5_H_5_)_2_, Mg(C_5_H_5_)_2_, Et Transitions De Phases De (C_4_H_4_)N)Fe(C_5_H_5_). Mol. Cryst. Liq. Cryst..

[45] Chhor K., Pommier C. (1984). Low temperature thermodynamic study of the stable and metastable phases of (C_4_H_4_N)Fe(C_5_H_5_). J. Chem. Thermodyn..

[46] Gregory J.K., Clary D.C., Liu K., Brown M.G., Saykally R.J. (1997). The Water Dipole Moment in Water Clusters. Science..

[47] Silvestrelli P.L., Parrinello M. (1999). Water Molecule Dipole in the Gas and in the Liquid Phase. Phys. Rev. Lett..

[48] Shieh J., Yeh J.H., Shu Y.C., Yen J.H. (2009). Hysteresis behaviors of barium titanate single crystals based on the operation of multiple 90° switching systems. Mater. Sci. Eng. B.

[49] Grimme S. (2006). Semiempirical GGA-type density functional constructed with a long-range dispersion correction. J. Comput. Chem..

[50] Sabatini R., Gorni T., de Gironcoli S. (2013). Nonlocal van der Waals density functional made simple and efficient. Phys. Rev. B..

[51] Vydrov O.A., Van Voorhis T. (2010). Nonlocal van der Waals density functional: The simpler the better. J. Chem. Phys..

[52] Thonhauser T., Zuluaga S., Arter C.A., Berland K., Schroder E., Hyldgaard P. (2015). Spin Signature of Nonlocal Correlation Binding in Metal-Organic Frameworks. Phys. Rev. Lett..

[53] Thonhauser T., Cooper V.R., Li S., Puzder A., Hyldgaard P., Langreth D.C. (2007). Van der Waals density functional: Self-consistent potential and the nature of the van der Waals bond. Phys. Rev. B.

[54] Langreth D.C., Dion M., Rydberg H., Schröder E., Hyldgaard P., Lundqvist B.I. (2005). Van der Waals density functional theory with applications. Int. J. Quantum Chem..

[55] Murray E.D., Lee K., Langreth D.C. (2009). Investigation of Exchange Energy Density Functional Accuracy for Interacting Molecules. J. Chem. Theory Comput..

[56] Lee K., Murray E.D., Kong L., Lundquist B.I., Langreth D.C. (2010). Higher-accuracy van der Waals density functional. Phys. Rev. B..

[57] Perdew J.P., Burke K., Ernzerhof M. (1996). Generalized Gradient Approximation Made Simple. Phys. Rev. Lett..

[58] Garrity K.F., Bennett J.W., Rabe K.M., Vanderbilt D. (2014). Pseudopotentials for high-throughput DFT calculations. Comput. Mater. Sci..

